# Nurse-led emergency department avoidance model of care for patients receiving cancer therapy in the ambulatory setting: a health service improvement initiative

**DOI:** 10.1186/s12913-023-09693-0

**Published:** 2023-06-29

**Authors:** Angela Mellerick, Georgina Akers, Niall Tebbutt, Tyler Lane, Rebecca Jarden, Kathryn Whitfield

**Affiliations:** 1 Olivia Newton John Cancer Centre, Studley Road, Heidelberg, VIC 3084 Australia; 2 Victorian Government Department of Health, Victoria, Australia; 3 Austin Health, Heidelberg, VIC Australia; 4 The University of Melbourne, Melbourne, VIC Australia; 5 School of Public Health and Preventive Medicine, Monash University, Melbourne, Australia; 6 Victorian Cancer Registry, Cancer Council Victoria, Melbourne, VIC Australia

**Keywords:** Cancer symptoms, Emergency department avoidance, Nurse-led clinic, Side effects, Supportive care, Systemic cancer therapy

## Abstract

**Aims:**

The Symptom and Urgent Review Clinic was a service improvement initiative, which consisted of the implementation and evaluation of a nurse-led emergency department (ED) avoidance model of care. The clinic was developed for patients experiencing symptoms associated with systemic anti-cancer therapy in ambulatory cancer settings.

**Methods:**

The clinic was implemented in four health services in Melbourne, Australia across a six-month period in 2018. Evaluation was by prospective data collection of the frequency and characteristics of patients who used the service, pre- and post-survey of patient reported experience, and a post-implementation survey of clinician engagement and experience.

**Results:**

There were 3095 patient encounters in the six-month implementation period; 136 patients were directly admitted to inpatient healthcare services after clinic utilization. Of patients who contacted SURC (*n* = 2174), a quarter (*n* = 553) stated they would have otherwise presented to the emergency department and 51% (*n* = 1108) reported they would have otherwise called the Day Oncology Unit. After implementation, more patients reported having a dedicated point of contact (OR 14.3; 95% CI 5.8–37.7) and ease of contacting the nurse (OR 5.5; 95% CI 2.6–12.1). Clinician reported experience and engagement with the clinic was highly favorable.

**Conclusion:**

The nurse-led emergency department avoidance model of care addressed a gap in service delivery, while optimizing service utilization by reducing ED presentations. Patients reported improved levels of satisfaction with ease of access to a dedicated nurse and advice provided.

## Introduction

The global incidence of cancer is increasing [1]. Expanding treatment options have resulted in cancer being considered a chronic disease. Coupled with an ageing patient population, this increases the complexity in the management of people with cancer [2]. While cancer treatment delivered within the ambulatory setting is cost effective and patient-centered, this places substantial burden on patients and carers to be knowledgeable and proactive in the recognition and management of treatment related side effects and cancer symptoms [3]. Physical, psychosocial and emotional side effects are common [4]. The organizational impact includes an increase in unplanned emergency department (ED) presentations [5]. The reasons for unplanned use of ED by cancer patients is multi-factorial and includes seeking reassurance from medical specialists based on a perception that ED can provide a higher standard of care than other settings, seeking treatment for symptoms according to clinician advice such as for fever [6], and because ED is the point of entry for inpatient care [7, 8].

As part of the consent process for chemotherapy treatment, clinical staff must provide information during discussion of the treatment plan. Patient recall is often limited and understanding of possible side effects and how they might self-manage them can be poor [9]. Patients may experience side effects in ways which impact patient quality of life and can result in escalation of symptoms that require medical management. Management of cancer symptoms and chemotherapy related toxicities sits within the core skillset of cancer nurses, particularly those working within the ambulatory setting [10]. Nurse-led models of care targeted to provide cancer patients and carers with knowledge to support self-care and self-management of symptoms can reduce symptom distress and severity [11, 12]. The more successful models include systematic use of telephone triage, extended practice hours and standardized treatment pathways [1]. Daly, Michaelis [12] described strategies to reduce acute care for patients which include identifying patients at high risk for unplanned acute care, enhancing access and care coordination, standardized clinical pathways for symptom management, developing urgent cancer care tactics, and early referral to palliative care. These models became more relevant during the COVID-19 pandemic when expert advice was that people with cancer were more susceptible to contracting COVID-19 and patients who did contract COVID-19 more likely to have clinical deterioration and severe adverse events [13]. The SURC model provides an opportunity to manage patients remotely reducing the risk of exposure to COVID-19.

The United Kingdom Oncology Nursing Society (UKONS) 24 Hour triage – rapid assessment toolkit provides a framework for assessment and standardized pathways according to patient risk. Patient assessment using this tool ensures patients with urgent care needs are identified and appropriate action is taken. For instance, patients assessed as lower-risk may be managed remotely, reducing unnecessary healthcare use. Conversely, patients assessed at higher-risk may be advised to attend the SURC for further assessment and intervention. The UKONS tool provides a structure for triage training and competency assessment of practitioners to ensure consistency of patient assessment and provision of advice [14].

### The Symptom and Urgent Review Clinic (SURC)

In 2013, the Symptom and Urgent Review Clinic (SURC) was piloted as a model of care in Western Health (WH), a healthcare service in Victoria, Australia, which later embedded it as the standard of care [15, 16]. The SURC model was designed to deliver the following: chemotherapy and self-care education to patients prior to treatment, a dedicated telephone line for patient assessment and management during treatment, and a dedicated physical space for nurses to assess patients with medical support for patient presentations outside the nursing scope. The UKONS assessment toolkit was a central tenet of this model, facilitating standardized treatment pathways for patients reporting toxicities associated with systemic cancer treatment [15, 16]. In 2018, the Victorian Department of Health identified the SURC model of care as a service that could more broadly be adopted by Victorian healthcare organizations to meet the local need to support patients receiving systemic anti-cancer therapy (SACT) and improve their experience of treatment and care [17]. What was not yet known, was whether patients and clinicians would engage with, and feel satisfied with the experience of the SURC.

The purpose of this study was to determine the frequency and characteristics of patients using the Symptom and Urgent Review Clinic (SURC), and patient and clinician engagement and experience with the SURC. To this end, there were five research questions (RQs):

RQ1: What is the frequency of patients using SURC?

RQ2: What are the characteristics of patients who use SURC?

RQ3: What is the patient reported experience pre- and post-SURC implementation?

RQ4: What is the clinician engagement with SURC?

RQ5: What is the clinician experience with SURC?

## Methods

### Setting

The SURC model of care was implemented and evaluated across four health services in metropolitan Melbourne, Victoria, Australia, where systemic anti-cancer therapy (SACT) was delivered in the ambulatory setting. The settings were Day Oncology Units (DOU) in two large metropolitan hospitals, a satellite unit in an outer metropolitan hospital, and a large pediatric cancer service. The four sites adapted SURC to local needs. Site A determined that nurse coordinators of the hematology service could provide support to these patients and established a dedicated oncology SURC. Site B elected to include a dedicated pharmacist one day a week, specifically to meet the needs of patients receiving oral chemotherapy agents. Site C included all tumor streams in their model. The pediatric site (D) elected for an alternative descriptor “Oncology Fast Track Clinic” [18]. There were no adverse patient outcomes reported across the four health services involved in this evaluation. All data were collected between April and September 2018.

### Study design

This service improvement evaluation included the implementation of the SURC within local organizations and prospective mixed-methods study design including 1) observing the frequency and characteristics of patients who used the service (RQ1 & 2), 2) pre- and post- survey of patient reported experience (RQ3), 3) survey of clinician engagement (RQ4) and experience (RQ5). The service improvement evaluation is reported according to the Revised Standards for Quality Improvement Reporting Excellence (SQUIRE 2.0; 19].

### Recruitment and participants

Patient participant recruitment for the patient survey was based on existing service use. The researchers predicted 146 new patient referrals per month and established a target sample of 74 patients at pre-implementation and 74 patients post-implementation. Patients receiving treatment in day oncology units across the three adult health services were provided a participant information and consent form. Those patients who provided consent were recruited into one of two surveys depending on the time of treatment. The first sample (pre-implementation) received treatment between February – April 2018 (*n* = 76). The second sample (post-implementation) received treatment between July – September 2018 (*n* = 76). All patients were receiving SACT and were identified based on chemotherapy activity (receiving cycle 2 or 3 of chemotherapy) and major tumor streams. The clinician participants (*n* = 137) were a convenience sample of clinicians who responded to email distribution of the clinician engagement survey tool. These clinicians were nursing, medical and allied health staff across cancer services and ED.

### Evaluation tools

#### Patient characteristics (RQ1&2)

A purpose-designed database captured frequency, patient demographic, disease and treatment-related information and SURC encounters, including patient education, telephone triage and physical presentation. The database included fields from the UKONS triage tool to enable patient toxicities to be graded according to the common terminology criteria (CTC) [20], and whether a medical review or inpatient admission was required. Patients who contacted SURC were also asked what action they would have taken if SURC was not available.

#### Patient experience (RQ3)

Patient experience before and after SURC was assessed using a researcher-developed and purpose designed survey administered via electronic tablet devices. Items included perceptions of education, decision support and overall experience in the Day Oncology Unit. The survey tool was developed, reviewed and tested in consultation with a panel of clinicians and a consumer representative. Patients were surveyed immediately before and six months after SURC implementation to determine if there was a perceived improvement in support after SURC implementation. Survey questions related to treatment and education were selected from the Victorian cancer patient experience survey [21] and are available at Open Science Framework [22]. Patients responded to either Likert scale or binary response questions. Likert scales had either a 4- or 5-point scale, where a rating of 1 was favorable. The pediatric site (site D) conducted its own survey which is reported elsewhere [18].

#### Clinician experience and engagement (RQ4&5)

A researcher-developed and purpose-designed clinician experience and engagement survey was developed and tested in consultation with a panel of clinicians. The survey was distributed across the three adult sites via SurveyMonkey™ four-months post SURC implementation. The intent of the clinician engagement survey was to determine clinician acceptability of the SURC model of care. Clinicans were asked 1) “If SURC wasn’t available what would you have done?” with the following response options: “Sent the patient to ED”, “Arranged a direct admission”, “Referred the patient to their GP”, “Other”; 2) “Were you happy with the care that was provided to the patient/s in the SURC?”, with dichotomous “Yes” or “No” response options, 3) “What impact do you believe the SURC program has had on patients receiving SACT in the ambulatory setting?”, with the following response options: “Favourable”, “Unfavourable” or “Neutral”, and 4) “Based on your experience, how would you improve the SURC model?”, which was a free response question. Site C was asked one further free response question 5) “If SURC was no longer available how would this impact you?”.

### Procedure

#### Phase one: implementation

The project was guided by a project governance committee comprising Victorian Department of Health stakeholders and medical, nursing, allied health, consumer representation from the evaluation sites, following Plan-Do-Study-Act (PDSA) methodology [23]. A project manager was appointed to coordinate the program and support the four sites to implement local models. The SURC model was based on the 24 Hour Triage model of the United Kingdom Oncology Nursing Society (UKONS) [14]. The model provided a structured approach to patient assessment of SACT-related toxicity. The rapid assessment toolkit provided a framework for assessment and standardized management pathways according to assessment of patient risk.

Communication skills training was delivered to support the development of advanced communication skills for nurses recruited to work in the SURC. This strengthened the SURC nurses’ skills to enable them to respond to the real-world patient presentations. In summary, the SURC model of care aimed to address the needs of patients receiving systemic anti-cancer therapy by providing 1) cancer treatment-specific information at the outset of treatment, 2) a single point of contact for patients to seek reassurance and advice for cancer symptom and toxicity management, and 3) a pathway into the organization for further assessment when warranted, including access to expert cancer medical staff. Each participating site was provided the standard patient assessment tools, communication skills training, and a reporting framework to support the standardization of assessment of patients and recording of service utilization.

#### Phase two: evaluation

An online portal was created to centralize evaluation resources, including the patient satisfaction survey tool, clinician engagement survey tool, and patient standardized assessment tools and pathways. Communities of practice events were convened every three months throughout the project. These enabled staff from the evaluation sites to share the enablers and barriers to implementation. Sites were required to provide formative reports at three, six, and nine-months to demonstrate project timelines were being met as well as a summative report at 12-months including service utilization data, patient and clinician satisfaction, and engagement survey results.

### Analysis

#### Quantitative data analysis

Patient characteristics and experience (RQ1-3)

Descriptive statistics using Microsoft® Excel® were used for the analysis of the frequency and characteristics of patients using SURC (RQs1&2). Patient reported experience responses (RQ3) were dichotomized based on whether the respondent gave the “best” response (“Yes I was given this information”, “Yes”, “Easy”, “Yes, Definitely”, “Confident”, “Completely Satisfied”) and negatively-framed questions were reversed to put all responses on a “worsened-improved” spectrum. Pre-post analyses were conducted in crude and adjusted logistic regression models. Covariates included patient sex and age group, whether they were born in Australia, spoke English as a preferred language, or lived with their carer. These analyses were conducted in R using RStudio (2020).

Clinician experience and engagement (RQ4&5)

Clinician experiences and engagement responses were predominantly binary “Yes”, “No” or “Favorable”, “Unfavorable” responses.

#### Qualitative data analysis

The two clinician free response questions were analyzed and reported following the six phase thematic analysis approach of Braun and Clarke [24]. The six phases included 1) familiarizing self with data, 2) generating initial codes, 3) searching for themes, 4) reviewing themes, 5) defining themes, and 6) producing the report. Two researchers independently coded the data and inductively developed the themes using either Microsoft® Excel® or an open card sort technique [25, 26]. After discussion, the researchers then met and agreed upon the themes before naming them. Pre-conceived researcher expectations were addressed reflexively throughout the evaluation from research questions to analysis.

### Ethical considerations

The evaluation protocol was approved by the Human Research Ethics Committee and organizational governance (Institutional Review Board of Monash Health RES-17-0000-635A). The evaluation methods including recruitment, data storage and confidentiality were conducted according to the research protocol. Informed consent was obtained from all participants in this evaluation.

## Results

### Frequency and characteristics of patients who used the service (RQ1&RQ2)

During the six-month evaluation period there were 3,095 SURC encounters from a total of 1,073 patients. The frequency and characteristics are reported in Table 1.

**Table 1:** Frequency and characteristics of patients who engaged with SURC.

	Site A Adult	Site B Adult	Site C Adult	Site D Pediatric	Overall
	*n* (%)	*n* (%)	*n* (%)	*n* (%)	*n* (%)
Total Encounters	975 (31%)	754 (24%)	518 (17%)	851 (27%)	3095
Individual Patients	363 (34%)	259 (24%)	229 (21%)	222 (21%)	1073
**SURC encounter by gender**					
Female	501 (51%)	435 (58%)	275 (53%)	364 (43%)	1574 (51%)
Male	474 (49%)	318 (42%)	243 (47%)	487 (57%)	1521 (49%)
**SURC encounter by treatment intent**					
Curative	277 (29%)	313 (42%)	54 (11%)	822 (97%)	1466 (47%)
Palliative	630 (65%)	423 (56%)	58 (11%)	29 (3%)	1140 (37%)
Not stated/Unknown	68 (6%)	17 (2%)	404 (78%)	0	489 (16%)
**SURC encounter by type**					
Phone triage-Incoming calls	393 (40%)	275 (37%)	279 (54%)	542 (64%)	1489 (48%)
Phone triage – Outgoing calls	170 (17%)	370 (49%)	65 (13%)	108 (12%)	713 (23%)
Pt Education	176 (18%)	≤ 5%	≤ 5%	≤ 5%	208 (7%)
SURC attendance	236 (24%)	77 (10%)	171 (33%)	201 (24%)	685 (22%)
**SURC encounter by tumor streams**					
Breast	226 (23%)	102 (14%)	66 (13%)	0 (0%)	394 (13%)
Colorectal	165 (17%)	282 (37%)	59 (11%)	0 (0%)	506 (16%)
Hematology	16 (2%)	0 (0%)	196 (38%)	412 (49%)	624 (20%)
Lung	191 (20%)	139 (19%)	73 (14%)	0 (0%)	403 (13%)
Pediatric Solid tumor	0 (0%)	0 (0%)	0 (0%)	297 (35%)	297 (10%)
Other	377 (38%)	230 (31%)	123 (24%)	142 (16%)	871 (28%)
**Physical SURC attendance (*n*** **= 685)**					
Medical Review Required	133 (56%)	18 (23%)	159 (93%)	130 (65%)	440 (64%)
Patients requiring Admission	26 (11%)	≤ 5%	61 (36%)	46 (23%)	136 (20%)

*Notes: Pt = patient; SURC = Symptom and Urgent Review Clinic; values under n = 5 are suppressed*

Of the 1,073 patients, 851 were from the adult sites and 222 from the pediatric site. There was variation in SURC presentations across the sites by gender, treatment intent, encounter type and tumor streams. More specifically, site A excluded hematology patients from their model and site B had a very limited hematology service. The pediatric center classified patients as solid tumor or hematology malignancy without specifying diagnosis.

While the initial design of the SURC model included pre-chemotherapy education, local adaptation at each site resulted in education being a central tenet to the model at site A alone. Importantly, however, almost a quarter of overall SURC activity (23%) consisted of outreach telephone calls made by SURC nurses to patients in the days following their first cycle of chemotherapy.

Medical review was required for 64% of patients who physically presented to SURC, predominantly for ordering diagnostics such as pathology and radiology, as well as prescribing. The proportion of patients requiring admission was variable across the sites and can most likely be attributed to the cohort of patients treated, particularly the number of hematology patients.

For each SURC encounter, a main presenting complaint was allocated to reflect the most distressing symptom or toxicity. The most commonly reported presenting patient complaints and their alternative action plans should SURC not have been available are reported in Table 2.

**Table 2:** Reasons for patients’ contact with SURC and alternative action plan

	n (%)
**Main presenting complaint**	
**Total encounters** ^**1**^	**2887**
Gastrointestinal issues	443 (15%)
Generally unwell	373 (13%)
Other specific chemo toxicity	321 (11%)
Non-clinical concern	297 (10%)
Pain	288 (10%)
Fever/sepsis	259 (9%)
Medication advice	232 (8%)
Diagnostics	196 (7%)
Respiratory issues	114 (4%)
Psychosocial issues	74 (3%)
Other	290 (10%)
**Alternative action if no SURC**	
**Total encounters** ^**2**^	**2174**
Called Day Unit	1108 (51%)
Presented to ED	553 (25%)
Done nothing	250 (12%)
Other	198 (9%)
Made a GP appointment	65 (3%)

Notes: ^1^ Excludes patient education encounters ^2^ Excludes patient education encounters and outgoing SURC calls; Abbreviations: ED = Emergency Department; GP = General Practitioner; SURC = Symptom and Urgent Review Clinic

Fever, pain, dehydration, gastrointestinal complaints and respiratory concerns were the leading causes of SURC presentation. Gastrointestinal symptoms including diarrhea, nausea, vomiting, anorexia and oral mucositis were the first reported symptom for 15% (*n* = 443) of encounters. Further, while the majority (70%) of patients with gastrointestinal toxicities were effectively managed via telephone triage, they also accounted for 83 (12%) of the 685 physical SURC presentations, one quarter (25%) of these patients reported they would have otherwise presented to ED.

Patients commonly experience multiple cancer symptoms and chemotherapy toxicities making it difficult for them to articulate a specific problem. Of those categorized as generally unwell, most were managed via telephone triage. Patients who physically presented to SURC as generally unwell reported higher frequency of fatigue compared to overall presentations and were more likely to report they would have otherwise presented to ED.

Pain was one of the leading reasons for SURC presentation. Of the 288 patients reporting pain as their primary complaint, 209 were via telephone triage. Of these 131 (63%) were able to be managed over the telephone. Medication advice was the most common outcome, often associated with arranging scripts to be faxed to local pharmacies and liaison with community-based palliative care teams. While fever accounted for 9% of the main presenting complaints across all sites, fever was considerably higher at sites C and D. This is reflective of the inclusion of hematology patients who receive more myelosuppressive regimens and experience more episodes of febrile neutropenia and sepsis. Of the 114 patients requiring physical attendance with fever/sepsis, 98 (86%) required medical review and 45% required direct admission to the ward. Of 114 patients reporting respiratory symptoms, 43 required physical presentation. Of these presentations, 37 (86%) required medical review and 16 (37%) were admitted for ongoing review and management. Of the 2174 patients who had a telephone triage or SURC attendance, in the event SURC was not available, 1108 (51%) said they would have contacted DOU and 553 (25%) said they would have presented to ED.

### Patient reported experience (RQ3)

**Fig. 1:**
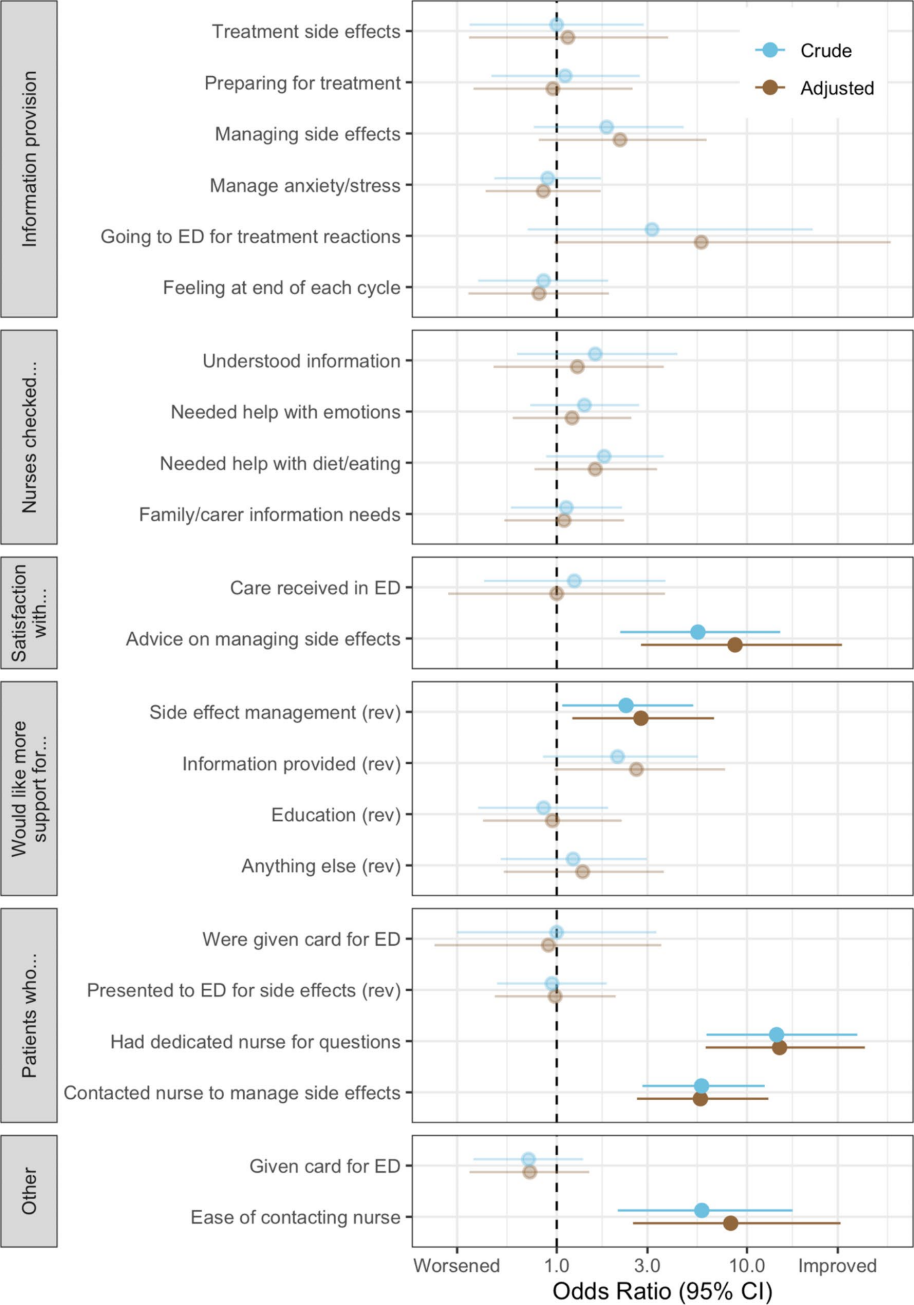
Patient reported experiences pre- and post-SURC implementation Notes: ED = Emergency Department; Faded data points are non-significant at p < 0.05. Questions reversed to a positive direction are noted with a “(rev)”

Of the adult patients, 152 were surveyed about their experience. Of the 22 outcomes measured, five were significantly different between the pre- and post-SURC implementation, illustrated in Fig. 1.

As associations were robust to adjustment for potential confounders, only adjusted Odds Ratios (OR) are reported here. Post-SURC, patients reported higher satisfaction with advice regarding management of side effects (OR: 8.66; 95% CI: 2.77–31.57), were more likely to report having a dedicated nurse they could contact (OR: 14.84; 95% CI: 6.06–41.62) and that they needed to contact a nurse for advice on managing side effects (OR: 5.69; 95% CI: 2.64–12.96), and it was easier to contact a nurse when needing advice (OR: 8.21; 95% CI: 2.52–31.01). They were also less likely to say that more support could have been provided regarding the management of cancer treatment side effects (OR: 2.77; 95% CI: 1.21–6.71); note that this outcome was reversed so that an OR > 1 is an improvement).

### Clinician experiences and engagement (RQ4 & RQ5)

There were 137 respondents to the clinician engagement survey across the three adult sites. Not all respondents answered all questions in the survey. In response to question (1) “If SURC wasn’t available what would you have done?”, of the 87 respondents, just over half of the clinicians (*n* = 50, 57%) responded that in the absence of SURC they would have referred the patient to ED. For question (2) “Were you happy with the care that was provided to the patient/s in the SURC?”, of the 89 respondents almost all (*n* = 88, 99%) of clinicians responded they were happy with the care delivered via the SURC model. For question (3) “What impact do you believe the SURC program has had on patients receiving SACT in the ambulatory setting?”, of the 118 respondents (*n* = 105), 89% of clinicians responded SURC had a favorable impact on patients receiving SACT in the ambulatory setting.

For question 4) “Based on your experience, how would you improve the SURC model?”, there were 25 participant responses from which three themes were identified. Theme one, ‘Knowledge and promotion of SURC’, included the clinician and patient knowledge of the availability and referral process to the service, for example, “More education about service and referral process and availability” and “More awareness. I don’t recall any education being given in ED and I think we only spread the word amongst ourselves a few months ago…”. Theme two, ‘Extended scope of SURC’, included the clinicians’ requesting an extension of the service to different patient cohorts such as hematology, for example, “to be available to all patients having treatment in haem & oncology settings” and “I would love it to be able to support in-patients in discharge planning, e,g,. fluid support/transition to discharge”. Theme three, ‘Extended hours of SURC’, included clinicians’ suggestions the service be available for longer periods of time. These suggestions were linked to statements where SURC may support ED avoidance, for example, “I would extend the hours - from 8am, until later in the evening, reflective of the busy periods in ED. I think the patient management is much better in SURC than in ED for most oncological issues” and “Extend hours. Create a larger space where managing multiple patients at one time would be much easier. Interface the service better with the inpatient units i.e., implement the triage tool to guide assessment and advice out of hours”.

For question 5) “If SURC was no longer available how would this impact you?”, 51 responses were used to derive three themes. Theme one, ‘increased pressure within the day oncology unit’, included the impact on the efficiency of the day oncology unit, increased pressure on nursing staff taking calls and managing unwell patients. For example “Significantly detrimental impact – as SURC improves flow of day oncology, improves managment of outpatients undergoing treatment, improves timeliness and appropriate care of unwell patients requiring direct admission, and also by early intervention decreases potential admissions altogether”. Theme two, ‘more referrals to ED’, included an increased volume of patients directed to ED, and the lack of cancer knowledge and skillset amongst ED staff. For example “We would have to send the patients to ED where they may or may not get an oncology experienced Doctor, and their time spent in ED seems to be longer, also SURC clinic have the skills to access CVADs to easily take required blood tests, causing less discomfort to the [patient]”. Theme three, ‘impact on the patient experience’, included the value to patients of knowing there is a cancer specialist that could be easily contacted and help them navigate their care. For example “More patients would have to spend longer time waiting in ED for assessment whereas SURC can often manage symptoms in this setting requiring less inpatient stays” and “No longer able to refer patients to SURC. I wouldn’t be able to give patients the peace of mind they get from knowing about SURC”.

## Discussion

Increasing health care costs, an ageing population and an increased incidence of cancer require novel models of care to be developed. The present study described the implementation and evaluation of SURC, a nurse-led model of cancer patient care in the ambulatory setting. Traditional models of care delivery in hospitals require all unplanned patient encounters to be triaged in the ED. In the six-month SURC implementation period, the 20% of patients who were directly referred (and subsequently admitted) to inpatient services from a physical attendance with the SURC represents 136 patients who didn’t attend ED. From a health service perspective, ED presentations by cancer patients place an additional burden on an already overcrowded ED [27]. Management of cancer patients within the ED leads to delays to provision of care, can be exhausting for patients and families, and poses significant patient risk [7]. The SURC model includes direct access to oncology medical staff for presentations that fall outside the nursing scope which mitigates against the delays and frustrations commonly described by patients presenting to the ED [8]. Without a dedicated resource, patients commonly contact the day oncology unit which is not resourced to manage unplanned care and lacks a pathway to appropriately triage and manage patient presentations [28]. Clinician feedback highlighted that SURC alleviated the day oncology unit workload.

During the implementation there were 3095 patient encounters across the four sites, showing that this model potentially addressed a gap in current service delivery. Gastrointestinal symptoms, feeling generally unwell, fever, pain, and respiratory symptoms were some of the leading reasons for SURC encounters. This is consistent with findings reported in the literature that these are among the most frequent reasons for ED presentation [13, 29, 30]. Symptoms leading to ED presentation often develop over several days, with earlier intervention potentially preventing emergency care [29]. The telephone triage may be one example of this earlier intervention as demonstrated in the current study where 25% of patients may have presented to ED if SURC had not been available.

For patients who reported being ‘generally unwell’, fatigue was a consistent feature. Managing patients presenting with fatigue associated with systemic anti-cancer therapy requires a specialized skillset. Specialist cancer nurses have the skillset and experience to determine if there are contributing factors, rule out or manage etiology such as direct tumor effect, sepsis, anemia, insomnia, concurrent medications, psychological strain and provide patients with strategies to deal with fatigue, such as exercise and psychosocial interventions [29, 31]. ED staff are not necessarily best positioned to determine the etiology or best management of these symptoms [30] and ED staff have identified multiple barriers to meeting the needs of patients with advanced cancer in the ED setting [8]. Nurse-led emergency department avoidance models of care for patients with cancer, such as remote management via telehealth for gastrointestinal symptoms, have been described as successful [32], and associated with reduced incidence of problems such as constipation and insomnia [33, 34].

The SURC model potentially addresses many of the gaps in the current healthcare system for patients who are expected to manage multiple, predictable symptoms and side-effects by providing timely access to expert advice and creating an alternate process for patients requiring unplanned admissions that avoids ED presentations. The majority (71%) of patients were managed via telephone triage and 25% of patients reported that in the absence of SURC they would have attended ED. Both patient and clinician satisfaction with SURC was high. Patients reported satisfaction with ease of access to a dedicated nurse and advice provided. These findings highlight the potential for SURC to prevent unplanned ED presentations and adds support to the model’s favorable economic outcomes [35]. Further benefits of proactive symptom management may be in the reduction of treatment delays and cancellations which could lead to sub-optimal disease outcomes [34].

### Strengths and limitations

A strength of this service improvement project was the use of multiple data sources to evaluate the effect of SURC. With only 152 respondents, the patient survey had limited statistical power to detect other benefits, or potential negative effects. The use of convenience sampling increased the risk of respondent bias. The clinician engagement survey was distributed via an email, which could have biased participation by only engaging those aware of SURC. The short evaluation period and limited resourcing reduced the number of data and time points that could be collected. As such it was not possible to match day oncology unit activity data in terms of tumor streams and demographic information thus it is not clear whether tumor streams are equally represented in SURC presentations. Heterogeneity in terms of patient cohort and operationalization of SURC at each site, including the culture of educating patients to contact the DOU would have influenced patient responses. A further limitation of this evaluation is that it did not measure ED presentations for patients with cancer pre- and post-implementation but used surrogate measures instead. This could be a potential area for future research.

## Conclusions

The frequency and characteristics of patient utilization of the service suggests the SURC model may address an unmet need for support in patients receiving systemic anti-cancer therapy in the ambulatory setting. The study identified several improvements in patient and clinician engagement and experience and found no evidence of negative effects. The SURC model may therefore offer a more appropriate model of care for people with cancer experiencing cancer symptoms and treatment related toxicities as an alternative to ED presentation.

## Data Availability

All data generated or analyzed during this study are included in this published article.

## List of abbreviations

CTC: Common Toxicity Criteria
DOU: Day Oncology Unit
ED: Emergency Department
GP: General Practitioner
PDSA: Plan, Do, Study, Act
Pt: Patient
RQ: Research Question
SACT: Systemic Anti-cancer Therapy
SQUIRE: Standards for QUality ImpRovement Excellence
SURC: Symptom and Urgent Review Clinic
UKONS: United Kingdom Oncology Nurses Society
